# A Practical Compendium of Midwifery

**Published:** 1850-01

**Authors:** 


*A Practical Compendium of Midwifery; being the compendium of Lectures on Midwifery, and on the Diseases of Women and Infants, delivered at St. Bartholomews Hospital, by the late* ROBERT GOOCH, M. D. Prepared for publication by GEORGE SKINNER, M. R. C. S. L. Fourth American edition. Philadelphia; Edmond Barrington and George D. Haswell; 1849. 8vo. pp.339.

The lectures of the late Dr. Gooch, although, in some respects, in the rear of the present position of Obstetrical science, will continue to be read with interest and profit. The practitioner may derive from the work much information, the value of which has not been impaired by *time*—that analytical agent which, in its relations to medical literature, is somewhat analogous to *destructive distillation* in chemistry. Aside from the matter, the style of Dr. Gooch's writings render them eminently readable.

Sold by G. H. Derby and Co.

